# Synergistic Effect of β-Cryptoxanthin and Epigallocatechin Gallate on Obesity Reduction

**DOI:** 10.3390/nu16142344

**Published:** 2024-07-20

**Authors:** Kazuhiko Nakadate, Kiyoharu Kawakami, Noriko Yamazaki

**Affiliations:** 1 Department of Functional Morphology, Meiji Pharmaceutical University, 2-522-1 Noshio, Kiyose, Tokyo 204-8588, Japan; k-kawakami@my-pharm.ac.jp; 2 Department of Community Health Care and Sciences, Meiji Pharmaceutical University, 2-522-1 Noshio, Kiyose, Tokyo 204-8588, Japan; nyamazak@my-pharm.ac.jp

**Keywords:** β-cryptoxanthin, green tea, gallate-type catechin, epigallocatechin gallate, obesity, high-calorie

## Abstract

Chronic obesity is an alarmingly growing global public health concern, posing substantial challenges for the prevention of chronic diseases, including hyperinsulinemia, type 2 diabetes, hyperlipidemia, hypertension, and coronary artery disease, and there is an urgent need for early mitigation strategies. We previously reported the obesity-reducing effects of green tea and β-cryptoxanthin intake. However, since tea has a complex mixture of compounds, it remained unclear which component contributed the most to this effect. Using high-performance liquid chromatography, we analyzed the components of tea in this study to determine if consumption of any combination of these compounds with β-cryptoxanthin had an obesity-reducing effect. Consuming epigallocatechin gallate (EGCG), a component of green tea, and β-cryptoxanthin for 4 weeks led to a decrease in body weight. Moreover, the weight and size of the white adipose tissues were significantly reduced, and blood biochemistry test results were comparable to normal values, with particular improvement in liver function. This indicated that intake of EGCG and β-cryptoxanthin reduces obesity in both subcutaneous and visceral fat. These findings suggest that simultaneous intake of EGCG and β-cryptoxanthin not only reduces obesity but also has a systemic beneficial effect on the body’s normal physiological function.

## 1. Introduction

The epidemic of overweight and obesity poses a serious challenge to chronic disease prevention and global health [1,2]. Over the past 40 years, the prevalence of obesity has more than doubled in many countries owing to factors such as economic growth, industrialization, motorized transportation, urbanization, sedentary lifestyles, and a shift toward consumption of processed food and high-calorie diets [3,4]. Chronic obesity is a direct cause of incident cardiovascular risk factors, such as hypertension, dyslipidemia, kidney disease, type 2 diabetes, and sleep disorders. Obesity is also a major risk factor for various cancer types, including breast and colorectal cancer. In addition, it can increase the risk of cancer recurrence and mortality among cancer survivors. Consequently, effective prevention and treatment strategies for obesity need to be developed [5,6,7]. 

The frequency of metabolic syndrome, which increases the risk of cardiovascular events and is associated with lifestyle-related diseases, is strongly associated with an increase in the incidence of obesity. Furthermore, the incidence of chronic liver diseases such as non-alcoholic fatty liver disease (NAFLD) and non-alcoholic steatohepatitis (NASH) in obese individuals is estimated to be approximately 33% [8]. NAFLD and NASH result from the excessive accumulation of fat in hepatocytes. This occurs when the rate of fatty acid uptake from the bloodstream and de novo synthesis exceeds the rate of fatty acid oxidation and export, leading to an excess of intrahepatic triglycerides. Increased levels of free fatty acids (FFA) are thought to promote inflammation, oxidative stress, and endoplasmic reticulum (ER) stress [9]. An association between adipose and muscle tissues has been suggested in the pathogenesis of liver diseases [10]. Adipose tissue secretes bioactive substances called adipokines, which play crucial roles in the pathogenesis of NAFLD [11,12]. Thus, associated disorders (type 2 diabetes mellitus, hypertension, and dyslipidemia) have an important role in the etiology of NAFLD.

In addition to clinical investigations in humans, several studies on chronic obesity have been performed using animal models. Post-natal administration of monosodium glutamate (MSG) has been used to induce chronic obesity in rodent models [13,14,15]. This model is very useful for analyzing the pathogenesis of chronic obesity since it does not require a long-term transition to a high-calorie diet; it induces chronic obesity by damaging the obesity center in the hypothalamus during post-natal development. Animal models of chronic obesity induced by MSG have been extensively used to investigate the intricate details of the pathophysiology of persistent obesity at both the cellular and tissue levels [16,17,18]. Chronic obesity in humans is often linked to high caloric intake, and thus, animal models of chronic obesity induced by high-calorie diets are more likely to replicate the pathogenesis of the condition in humans [19]. We have reported earlier the pathophysiology and improvement of chronic obesity in MSG-induced and high-fat diet-induced mouse models [16,19]. Each of these animal models has enabled histopathological and other investigations that are difficult to perform in humans.

Studies on the alleviation of chronic obesity and cohort studies with human participants have reported that green tea was involved in the reduction in fat absorption [2]. Catechins in green tea have been suggested to have an obesity-ameliorating effect and may have the potential to reduce chronic obesity [20]. Among the eight types of catechins found in tea, the presence of “gallated” catechins, such as epigallocatechin gallate (EGCG), epicatechin gallate (ECG), gallocatechin gallate, and catechin gallate, is believed to have the greatest impact on fat absorption. Additionally, the “free” catechins, including epigallocatechin (EGC), epicatechin (EC), gallocatechin, and catechin, are also thought to play a role in this process [20,21]. While these tea catechins are effective in alleviating obesity by reducing fat absorption, it has not yet been verified whether they efficiently metabolize accumulated lipids in the body during the pathogenesis of chronic obesity [22,23,24]. Furthermore, clinical studies in humans are challenging because it is difficult to sustain high concentrations of tea catechins for a long period [24]; studies on the benefits of combining tea catechins with other foods have not yet demonstrated effectiveness. We previously reported that β-cryptoxanthin, which is abundant in mandarins, is effective in augmenting the weight loss effect of green tea [16,19]. We have also reported that simultaneous consumption of these two can reduce obesity even with small amounts of green tea intake and are effective against various obesity-related diseases involving decreased liver function. However, the component of green tea responsible for the obesity-reducing effect has not been clarified.

In this study, we performed a detailed analysis of the tea catechins that are mainly effective in suppressing chronic obesity. We report the effects of simultaneous ingestion of a single tea catechin component, with its chronic obesity suppression effect, and β-cryptoxanthin, which we found to synergistically enhance the effect of tea catechin.

## 2. Materials and Methods

### 2.1. Animals

Male C57BL/6J mice were used in this study, as in our prior studies [16,19]. Fourteen female mice and their corresponding eight male pups were procured from Japan SLC Co. Ltd. (Tokyo, Japan) at post-natal day 0. The offspring were raised alongside their mothers in the same cage until they reached the age of 3 weeks, at which point they were weaned. Post-weaning, two mice per cage were maintained. The animals were reared in an environment with constant temperature (23 ± 2 °C), humidity (55 ± 10%), and light–dark cycle (12 h), as well as ad libitum access to water and food. 

The experimental protocol was sanctioned by the Laboratory Animal Ethics Committee at Meiji Pharmaceutical University (No. 2707, 1 April 2020–2024), and all procedures were executed in accordance with the Meiji Pharmaceutical University Experimental Animal Guidelines.

### 2.2. Generation of Chronically Obese Mice and Body Weight Measurement

In our previous study, we developed a high-calorie intake mouse model of chronic obesity [19]. High-calorie (HC) diets (D12492 ultra-high-fat feed with 60 kcal % fat content, EPS EKISHIN Co., Ltd., Tokyo, Japan) were provided from post-weaning until the last day of the experiment (total 104 mice). Control mice (*n* = 8) were fed a control diet (D12450B Research Diets for Control; EPS EKISHIN) (control group).

Following the protocol of our previous studies [16,18,19], all post-weaning mice were weighed daily at noon.

### 2.3. Measurement of Green Tea Catechins

The components of tea catechins and their amounts were measured using high-performance liquid chromatography (HPLC), a quantitative method that we used to eliminate substances other than catechins. After the selection of extraction solvents and manipulation methods, 50 mg of crushed tea leaves was extracted for 2 h in 1 mL 10% acetonitrile, followed by centrifugation and collection of the supernatant. Caffeine was not removed using this preparation method. The supernatant was passed through a Millipore filter (0.45 mm), and 2.7 mL was injected into an HPLC (e-HPLC Kotori type A, Uniflows Co., Ltd., Tokyo, Japan) column (PEGASIL ODS SP100; Senshu Kagaku Co., Ltd., Tokyo, Japan). The eluent comprised 87.45% DDW, 12.5% ethanol, and 0.05% acetic acid at a flow rate of 1.6 mL/min. Detection was performed using a UV detector at a wavelength of 265 nm. Standard solutions (Sigma-Aldrich, Tokyo, Japan) were used to determine the concentration of each catechin.

Five types of commercially available green tea leaves were used: ITO EN home size green tea (ITO EN, Co., LTD, Tokyo, Japan); ITO EN ajino Taiko-ban green tea (ITO EN, Co., Ltd., Tokyo, Japan); Ishidacyaya Shizuoka tea (Ishidacyaya, Co., Ltd., Shizuoka, Japan); Oigawa Chaen Green Tea (Oigawachaen, Co., Ltd., Shizuoka, Japan); and Juroen Yamecha (Juroen, Co., Ltd., Hiroshima, Japan). Three extractions were carried out for each type of tea leaf, and the average value was determined.

### 2.4. Administration of Green Tea Catechins and β-Cryptoxanthin

Following our earlier study [16,18,19], green tea (3.4 mg/kg), a half concentration of green tea (1.7 mg/kg), a half concentration of each of green tea catechins (EGC; 0.030 mg/kg, EGCG; 0.067 mg/kg, EC; 0.007 mg/kg, ECG; 0.007 mg/kg, Sigma-Aldrich Japan, Tokyo, Japan), and β-cryptoxanthin (50 mg/kg body weight, Wako Pure Chemical Industries Ltd., Tokyo, Japan), which were identified and isolated using HPLC, were administered to the animals orally for 4 weeks, starting from week 11 and ending at week 15 of their age (*n* = 8/each group).

### 2.5. Blood Biochemistry Analysis

After a period of 15 weeks, blood samples were obtained from the tail vein of each mouse (*n* = 8 per group) in 1.5 mL microcentrifuge tubes. These tubes were allowed to stand for 30 min and centrifuged at 1500× *g* for 20 min to separate the sera. These sera were quickly frozen and stored until further experimental analysis. 

Blood glucose, total protein, total lipid, low-density lipoprotein (LDL) cholesterol, high-density lipoprotein (HDL) cholesterol, free cholesterol, free fatty acid (non-esterified fatty acid; NEFA), triglycerides (TG; neutral fat), alkaline phosphatase (ALP), aspartate aminotransferase (AST), and alanine aminotransferase (ALT) levels were measured using appropriate test kits (Wako Pure Chemical Industries Ltd., Osaka, Japan).

### 2.6. Histological Analysis

After obtaining the blood samples, the mice (*n* = 8/each group) were sedated using 5% isoflurane, and their internal adipose tissues were rapidly collected and weighed. The white adipose tissues were then immersed in 4% paraformaldehyde solution (pH 7.4) for 2 days. They were then trimmed to reasonable sizes, washed in 0.1 M phosphate buffer, and dehydrated with a 50 to 100% gradient of ethanol. Following the addition of Lemosol A (Wako Pure Chemical Industries Ltd., Tokyo, Japan), the samples were embedded in paraffin wax. Sections of paraffin blocks were sliced to a thickness of 5 μm using a sliding microtome (model REM-710, manufactured by Yamato Kohki Industrial, Tokyo, Japan) and stained with hematoxylin–eosin stain (Muto Pure Chemicals Co. Ltd., Tokyo, Japan). The sections were then dehydrated and covered with coverslips. All sections were meticulously examined using an optical microscope (BZ-X700; Keyence, Osaka, Japan), and high-quality images were captured. The ImageJ software (version 1.46) was utilized to analyze the area of white adipose cells, resulting in precise measurements. Five images were randomly taken per animal, and the area of all white adipose cells was measured. An average of 300 cells were measured per animal. 

### 2.7. Statistical Analyses

Weight changes were analyzed at 11 and 15 weeks of age and were averaged for each group. All the values obtained from the blood tests and histological analysis were tabulated for each group. 

Data obtained in this study are presented as the mean ± standard deviation. The statistical significance of comparisons was determined using analysis of variance, with a *p*-value of less than 0.05 indicating a significant difference. Statistical analyses were performed using Microsoft Excel and StatView statistical software (version 5.0.1, SAS Institute Inc., Cary, NC, USA).

## 3. Results

### 3.1. Measurement of Catechin Concentration

**Figure 1:**
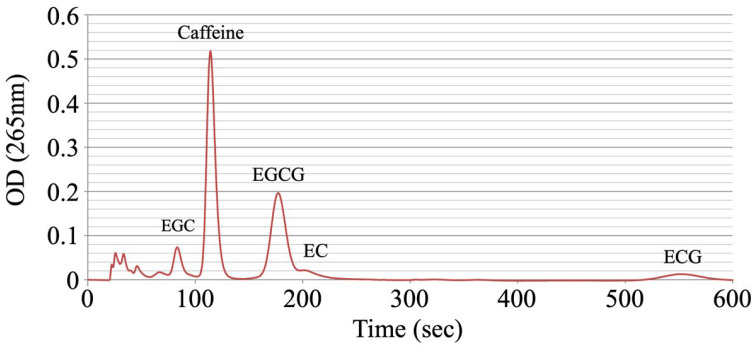
High-performance liquid chromatography profile of a green tea sample showing the different catechins. EGC: epigallocatechin; EGCG: epigallocatechin gallate; EC: epicatechin; ECG: epicatechin gallate; OD: optical density.

The concentration of each catechin in tea was measured using HPLC. In this study, five commonly available green teas were used to measure catechins; the profile of one of the samples is shown in Figure 1, where the four major catechins are identified. The concentration of catechins in each tea leaf was calculated from the concentration of the standard solution. The average values of three extractions from each green tea leaf type are shown in Table 1, together with the total average values of the five types. EGCG and EGC were found in high concentrations, while EC and ECG were found in relatively low concentrations.

### 3.2. Weight Change Associated with Intake of Each Catechin and β-Cryptoxanthin

The effects of weight reduction are summarized: Significant weight gain was observed in HC-fed mice compared to normal diet-fed mice. Green tea and β-cryptoxanthin alone did not show weight-reducing effects. Significant weight loss was observed with the simultaneous intake of green tea and β-cryptoxanthin, and this reduction was also observed with the simultaneous intake of EGCG and β-cryptoxanthin. A detailed description is given below.

β-cryptoxanthin in combination with either green tea or each of the catechins identified using HPLC were ingested by the animals for 4 weeks, and body weight changes were measured (Table 2). HC-fed mice exhibited significant weight gain at 11 and 15 weeks of age compared to normal-reared mice. No significant weight changes were observed in mice receiving green tea, a half concentration of green tea, or β-cryptoxanthin for 4 weeks.

Similar to the findings of our previous study, animals that consumed both a half concentration of green tea, which contains all catechins, and β-cryptoxanthin showed significant weight loss compared to obese mice who consumed water. Our previous study has shown no significant weight loss with the same concentration of green tea alone, suggesting that this is a synergistic effect of β-cryptoxanthin [19]. Animals that consumed the HC diet and EGC, found in high concentrations in green tea, simultaneously showed no change in body weight compared to those fed solely an HC diet. On the other hand, a significant reduction in body weight gain was observed in the rodents who ingested EGCG and β-cryptoxanthin simultaneously. No significant variation in body weight gain was observed between the HC group and the mice that ingested EC, ECG, and β-cryptoxanthin. The effects of each tea catechin alone on body weight were further examined, but no significant weight loss was observed in all groups.

The summarized effects on adipose tissue are as follows: Significant white adipose tissue weight gain was observed in HC-fed mice compared to normal mice. The reduction in white adipose tissue effect was not observed with green tea alone or with β-cryptoxanthin alone. A significant weight loss was observed with a simultaneous intake of green tea and β-cryptoxanthin, and this loss was also observed with a simultaneous intake of EGCG and β-cryptoxanthin. A detailed description is given below.

Body adipose tissue weights of all animals were measured (Table 3). Animals given an HC diet displayed significantly more weight of white adipocytes than the control animals fed a normal diet. There was also no significant change in the weight of white adipocytes in the groups treated with green tea alone or β-cryptoxanthin alone. Conversely, animals fed half of the green tea and β-cryptoxanthin exhibited a statistically significant reduction in the weight of their white adipocyte tissue. Consistent with body weight data, the animals that ingested EGC or EC and β-cryptoxanthin did not show a significant decrease in adipose tissue weight, whereas those who ingested EGCG and β-cryptoxanthin showed a significant decrease in adipose tissue weight. Mice fed ECG and β-cryptoxanthin showed significant adipose tissue weight reduction compared to HC-fed mice. However, compared to the weight of adipose tissue in normal mice, it was still heavier, suggesting that the reduction effect was weak. The effects of each tea catechin alone on white adipocyte tissue weights were further examined, but no significant weight loss was observed in all groups.

Next, the size of white adipose cells was measured (Table 4). White adipose cells in mice fed a high-calorie diet were enlarged. A significant reduction in area was observed in mice that consumed green tea and β-cryptoxanthin simultaneously. A significant decrease in cell area was also observed in mice that consumed EGCG and β-cryptoxanthin. On the other hand, there was no significant decrease in cell area in the EGC and β-cryptoxanthin-, EC and β-cryptoxanthin-, and ECG and β-cryptoxanthin-ingested mice, respectively.

### 3.3. Blood Biochemistry Tests

**Figure 2:**
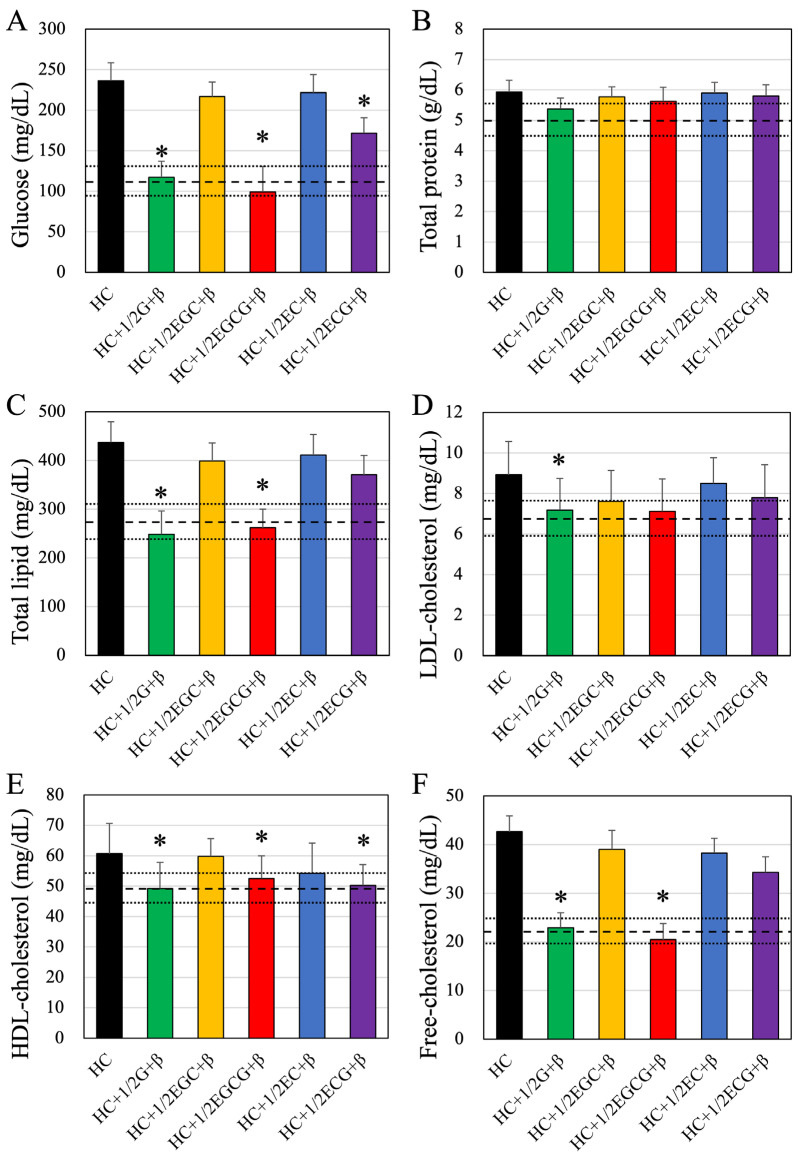

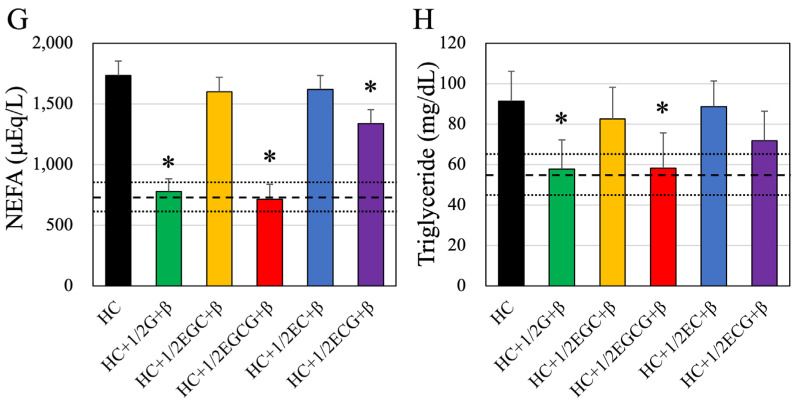
Blood biochemistry values of mice in each group. (**A**) Blood glucose levels; (**B**) Total protein levels; (**C**) Total lipid levels; (**D**) Low-density lipoprotein (LDL) cholesterol levels; (**E**) High-density lipoprotein (HDL) cholesterol levels; (**F**) Free cholesterol levels; (**G**) Free fatty acid (non-esterified fatty acid; NEFA) levels, and (**H**) Triglyceride (TG; neutral fat) levels. The dashed and dotted lines in each figure indicate the average and standard deviation values, respectively, of control mice. HC, HC + 1/2G + β, HC + 1/2EGC + β, HC + 1/2EGCG + β, HC + 1/2EC + β, and HC + 1/2ECG + β indicate the different combinations of diet administered to the animals. HC, high-calorie-fed; G, green tea; β, β-cryptoxanthin; EGC, epigallocatechin; EGCG, epigallocatechin gallate; EC, epicatechin; ECG, epicatechin gallate. Data are presented as mean ± standard deviation. * *p* < 0.05 compared to high-calorie-fed group.

Next, we analyzed the changes in blood parameters associated with the simultaneous ingestion of each of the tea catechins and β-cryptoxanthin by measuring the blood biochemical parameters (Figure 2 and Table 5). The effects on each hematology and biochemistry test item are summarized. Almost all the parameters were significantly worse in HC-fed mice than in normal mice. Green tea and β-cryptoxanthin did not significantly improve blood chemistry tests when consumed separately. However, when both were consumed in combination, significant improvements were seen. The improvement was also confirmed by simultaneous intake of EGCG and β-cryptoxanthin. A detailed description is given below.

Higher values were observed in mice fed a high-calorie diet (HC) compared to control mice raised on a normal diet (dashed line). Mice that ingested half of the green tea containing total catechins and β-cryptoxanthin (HC + 1/2G + β group) showed significant improvement, similar to normal values.

It can be observed that EGC and β-cryptoxanthin (HC + 1/2EGC + β group) and EC and β-cryptoxanthin (HC + 1/2EC + β group) did not show significant improvement, whereas EGCG and β-cryptoxanthin-fed mice (HC + 1/2EGCG + β group) and ECG and β-cryptoxanthin intake mice (HC + 1/2ECG + β group) showed significant lowering of glucose levels, similar to normal values (dashed line). On the other hand, no significant decrease in blood glucose levels was observed in the other groups treated alone (Table 5). The total proteins in serum showed no significant differences among all groups (Figure 2B and Table 5).

Regarding total lipids, only mice fed EGCG and β-cryptoxanthin (HC + 1/2EGCG + β group) showed significant improvement similar to the HC + 1/2G + β group, while in the other groups, there was no improvement (Figure 2C and Table 5). Analysis of blood LDL cholesterol levels (Figure 2D and Table 5) indicated no improvement in any of the groups except half of the green tea containing total catechins and β-cryptoxanthin (HC + 1/2G + β) group. Analysis of blood HDL cholesterol levels (Figure 2E and Table 5) showed that mice consuming EGCG and β-cryptoxanthin (HC + 1/2EGCG + β group) and ECG and β-cryptoxanthin (HC + 1/2ECG + β group) showed similar improvement as the HC + 1/2G + β group. Analysis of free blood cholesterol (Figure 2F and Table 5) showed significant improvement only in mice fed EGCG and β-cryptoxanthin (HC + 1/2EGCG + β group), similar to the HC + 1/2G + β group. NEFA (Figure 2G and Table 5) showed a significant improvement in mice fed EGCG and β-cryptoxanthin (HC + 1/2EGCG + β group), similar to the HC + 1/2G + β group. Mice fed ECG and β-cryptoxanthin (HC + 1/2ECG + β group) also showed significant improvement, although not to the level of control mice. Analysis of blood triglyceride levels (Figure 2H and Table 5) showed significant improvement only in mice fed EGCG and β-cryptoxanthin (HC + 1/2EGCG + β group), similar to the HC + 1/2G + β group.

### 3.4. Liver Function Tests

**Figure 3:**
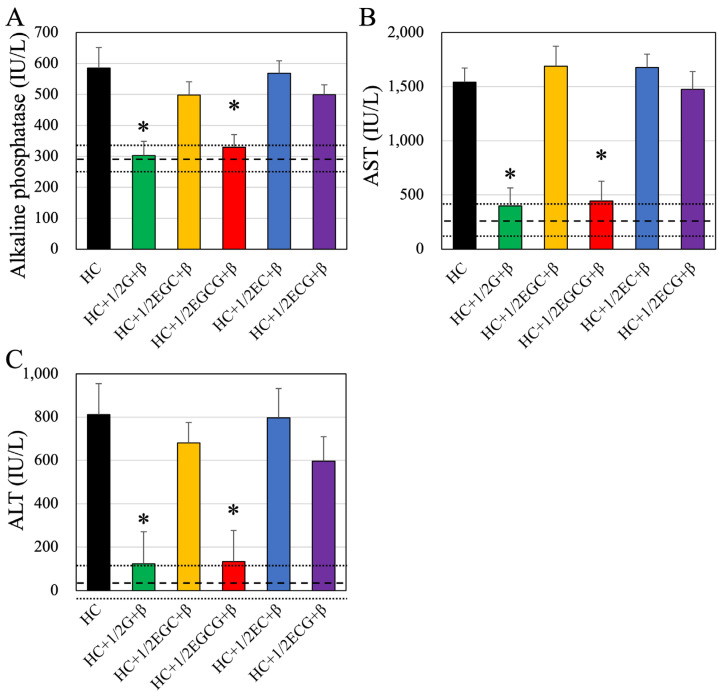
Blood values of (**A**) alkaline phosphatase (ALP); (**B**) aspartate aminotransferase (AST), and (**C**) alanine aminotransferase (ALT). Dashed and dotted lines in each figure indicate the average and standard deviation values, respectively, of control mice. HC, HC + 1/2G + β, HC + 1/2EGC + β, HC + 1/2EGCG + β, HC + 1/2EC + β, and HC + 1/2ECG + β indicate different combinations of diet administered to the animals. HC, high-calorie-fed; G, green tea; β, β-cryptoxanthin; EGC, epigallocatechin; EGCG, epigallocatechin gallate; EC, epicatechin; ECG, epicatechin gallate. Data are presented as mean ± standard deviation. * *p* < 0.05 compared to high-calorie-fed group.

In addition to the usual blood tests, liver function was assessed by measuring the following three parameters: ALP; AST; and ALT (Figure 3A–C and Table 6). The effects on each test parameter are summarized below. Almost all of the investigated parameters were significantly worsened in HC-fed mice compared to normal mice. The effects of green tea alone and β-cryptoxanthin alone on each test item were not observed, but significant improvement was observed when green tea and β-cryptoxanthin were ingested simultaneously. The improvement was also confirmed by simultaneous intake of EGCG and β-cryptoxanthin. A detailed description is given below. For these tests, similar results were obtained for all three combinations. Compared to control mice fed a normal diet (dashed line), mice fed a high-calorie diet (HC) showed higher values for all three parameters. Obese mice fed half (1.7 mg/kg) of green tea and β-cryptoxanthin (50 mg/kg) for 4 weeks (HC + 1/2G + β group) showed significant improvement in each of the parameters, similar to normal values. There was no improvement in the EGC and β-cryptoxanthin-fed mice, EC and β-cryptoxanthin-fed mice, and ECG and β-cryptoxanthin groups, while EGCG plus β-cryptoxanthin significantly decreased the values of each of these parameters to levels similar to those of control mice, indicating improvement in liver function. The effects of each tea catechin alone on liver function were further examined, but no significant reduction was observed in all groups (Table 6).

## 4. Discussion

Losing weight has been clinically proven to reduce the risk of several diseases. Weight loss is very easy in cases of diseases and hospitalization but very difficult in real-life situations. Exercise and diet improvement are essential for obesity prevention and weight reduction under the guidance of a physician. Effective performance of these activities can lead to weight loss and maintenance. However, many obese individuals struggle with these activities due to the inability to exercise or restrict their diet. Therefore, there is a need for foods that induce weight loss and can help prevent and improve diseases, especially lifestyle-related ones. Green tea, which has been enjoyed for a long time in many countries, is believed to aid in weight loss. Catechins, polyphenols abundant in green tea, are reported to suppress fat absorption when consumed with meals by inhibiting the activity of pancreatic lipase in the small intestine and promoting the excretion of fat in the stool [25,26]. Thus, green tea may be an effective dietary supplement for individuals attempting to regulate their body weight. Gallate catechins, which are present in tea, bind to bile acid micelles in the small intestine, resulting in the release of cholesterol from the micelles, which consequently reduces cholesterol absorption and blood cholesterol levels [27]. It is generally accepted that tea catechins, once absorbed into the body, are transported to their target cells and function as bioactive compounds [28]. Consumption of tea catechins has been found to increase the expression and activity of lipolytic enzymes in adipocytes, which, in turn, enhances the release of fat-derived glycerol [22]. Furthermore, research has demonstrated that they can enhance the functioning of enzymes that play a role in the β-oxidation process in the liver and skeletal muscles, as well as fatty acid transport enzymes in skeletal muscles. When used in conjunction with exercise, these compounds have been shown to increase overall β-oxidation activity [29]. Increasing fat oxidation and energy expenditure during daily activities can improve energy and lipid balance while also reducing visceral adiposity. Furthermore, recent studies have demonstrated that tea catechins can stimulate energy metabolism in brown adipose tissues, potentially leading to a decline in chronic obesity [30,31]. In addition, tea catechins, specifically gallate catechins in green tea, have a moderate effect on fat absorption [31,32]. Consistent with numerous studies, long-term intake of gallate catechins achieves a substantial reduction in both body weight and visceral adipose tissue [33]. In humans, however, it is necessary to consume at least 200 mg per day, equivalent to about five cups of green tea, to obtain the effect of obesity inhibition. Therefore, many people with obesity may not obtain sufficient obesity-inhibitory effects from green tea. To consume enough green tea and achieve the desired effect, commercially available supplements and green tea containing high concentrations of tea catechins may be procured; however, these products are not commonly accessible, and their uptake is limited by factors such as unpleasant taste and high product cost.

Therefore, synergistic consumption with foods other than tea is desired to obtain the maximum effect of the tea catechins in green tea. Our previous reports used mandarin oranges, which contain β-cryptoxanthin that is generally present in citrus fruits [16,19], which is expected to have stronger antioxidant effects than polyphenols. We have reported its relationship with various diseases, including preventive effects on chronic kidney disease. In this study, we found that β-cryptoxanthin and a single component of tea catechin (EGCG), taken together for 4 weeks, could have an anti-obesity effect with a lower intake of green tea catechin than previously reported. This amount can easily be consumed in a single day and is expected to help reduce obesity in real-life situations. The results of this study, which are mainly based on blood component analysis, are similar to our previous reports, suggesting various functional improvements, such as improved liver function and reduced adipose tissue. Previously, we conducted a cohort study in humans to examine the effect of food combinations comprising green tea and citrus-derived polyphenols [34]. In that cohort study, long-term (12 weeks) consumption was reported to result in weight loss, improved body mass index, and improved blood chemistry tests.

The present study has some limitations. This study was conducted using an extrapolated human–animal model. Extensive research has been conducted using animal models of human diseases, resulting in significant findings. However, it is crucial to acknowledge that these animal models are not identical to humans. Thus, it is important to recognize that the results obtained from animal models may not be directly applicable to humans. Furthermore, while green tea used in this study is commercially available, there are numerous other green tea products available in various countries. Therefore, future investigation is needed to determine if the green tea content is consistent across all green teas. Additionally, although tea leaves were used in this study, there are commercially available green tea products that facilitate more efficient extraction, such as granular and powdered products. It is possible that each of the ingredients in these products may have a greater weight-reducing effect, and the relationship between processing technologies for green tea is also an area for future research. In addition, the effects of green tea components on intestinal bacteria and intestinal cells were not investigated and are, thus, a subject for future investigation. However, based on the findings of the present study, many citrus-derived polyphenols may potentiate the action of green tea catechins in humans.

In the future, we believe that the identification of ingredients that potentiate the effects of tea catechins in reducing obesity will contribute to the prevention of obesity. The findings of this research are anticipated to contribute to a clearer understanding of the anti-obesity mechanism of tea catechins, as well as provide insights into the food combination effects of green tea catechins and citrus-derived ingredients. Based on the present study findings, future empirical studies on human subjects will provide valuable information to support further research in these areas.

## 5. Conclusions

In this study, we found that EGCG, one of tea catechins, and β-cryptoxanthin, contained in citrus fruits, have synergistic effects on obesity reduction. We also found that the amount that is effective for the anti-obesity effect can be easily ingested in our daily lives. These findings may contribute not only to the reduction in chronic obesity in humans but also to the development of nutraceuticals in the future.

## Figures and Tables

**Table 1:** Average concentration (mg/mL) of catechins and caffeine.

	EGC	Caffeine	EGCG	EC	ECG
No.1 Average	0.900	0.619	1.942	0.231	0.196
No.2 Average	1.050	0.583	1.833	0.192	0.174
No.3 Average	0.788	0.583	2.035	0.240	0.239
No.4 Average	0.775	0.631	1.987	0.183	0.250
No.5 Average	0.963	0.607	2.067	0.212	0.185
Total Average	0.895	0.605	1.973	0.212	0.209
S.D.	0.117	0.021	0.091	0.025	0.034

EGC: epigallocatechin; EGCG: epigallocatechin gallate; EC: epicatechin; ECG: epicatechin gallate; S.D.: standard deviation.

**Table 2:** Changes in the body weight (11–15 weeks age).

	11 Weeks (g)	15 Weeks (g)
Control	22.76 ± 1.69 *	28.84 ± 1.94 *
HC	31.26 ± 0.70 #	39.86 ± 1.86 #
HC + Green Tea	32.32 ± 1.35 #	36.02 ± 1.91 #
HC + 1/2 Green Tea	31.73 ± 1.60 #	39.03 ± 1.33 #
HC + β-cryptoxanthin	31.95 ± 1.36 #	39.98 ± 1.97 #
HC + 1/2 Green Tea + β-cryptoxanthin	31.66 ± 0.99 #	30.64 ± 2.13 *
HC + 1/2 EGC + β-cryptoxanthin	32.44 ± 0.84 #	39.98 ± 2.10 #
HC + 1/2 EGCG + β-cryptoxanthin	31.86 ± 0.84 #	29.58 ± 1.76 *
HC + 1/2 EC + β-cryptoxanthin	31.66 ± 0.99 #	39.70 ± 2.03 #
HC + 1/2 ECG + β-cryptoxanthin	31.86 ± 1.14 #	37.74 ± 1.17 #
HC + 1/2 EGC	32.19 ± 1.57 #	40.01 ± 1.47 #
HC + 1/2 EGCG	30.91 ± 1.23 #	37.92 ± 2.01 #
HC + 1/2 EC	32.14 ± 1.51 #	39.25 ± 1.35 #
HC + 1/2 ECG	31.35 ± 0.94 #	38.46 ± 2.14 #

HC: high-calorie-fed mice; EGC: epigallocatechin; EGCG: epigallocatechin gallate; EC: epicatechin; ECG: epicatechin gallate. #: *p* < 0.05 vs. age-matched control value. *: *p* < 0.05 vs. age-matched HC value.

**Table 3:** The weight of visceral white adipose tissue at 15 weeks of age.

	Weight (g)
Control	0.79 ± 0.31 *
HC	3.14 ± 0.41 #
HC + Green Tea	2.98 ± 0.40 #
HC + 1/2 Green Tea	3.21 ± 0.35 #
HC + β-cryptoxanthin	3.06 ± 0.42 #
HC + 1/2 Green Tea + β-cryptoxanthin	0.74 ± 0.42 *
HC + 1/2EGC + β-cryptoxanthin	2.88 ± 0.51 #
HC + 1/2 EGCG + β-cryptoxanthin	0.72 ± 0.49 *
HC + 1/2 EC + β-cryptoxanthin	2.79 ± 0.46 #
HC + 1/2 ECG + β-cryptoxanthin	1.74 ± 0.52 #*
HC + 1/2EGC	3.02 ± 0.43 #
HC + 1/2 EGCG	3.01 ± 0.38 #
HC + 1/2 EC	3.19 ± 0.36 #
HC + 1/2 ECG	3.00 ± 0.44 #

HC: high-calorie-fed mice; EGC: epigallocatechin; EGCG: epigallocatechin gallate; EC: epicatechin; ECG: epicatechin gallate. #: *p* < 0.05 vs. age-matched control value. *: *p* < 0.05 vs. age-matched HC value.

**Table 4:** Average area of adipocytes in the abdomen at 15 weeks of age.

	Area (mm^2^)
Control	728.75 ± 433.67 *
HC	1999.0 ± 1038.9 #
HC + Green Tea	1798.4 ± 582.41 #
HC + 1/2 Green Tea	1933.2 ± 701.39 #
HC + β-cryptoxanthin	2013.2 ± 821.92 #
HC + 1/2 Green Tea + β-cryptoxanthin	804.38 ± 448.30 *
HC + 1/2 EGC + β-cryptoxanthin	1721.5 ± 835.20 #
HC + 1/2 EGCG + β-cryptoxanthin	847.33 ± 398.32 *
HC + 1/2 EC + β-cryptoxanthin	1902.3 ± 964.12 #
HC + 1/2 ECG + β-cryptoxanthin	1418.4 ± 624.11 #
HC + 1/2 EGC	1897.3 ± 705.28 #
HC + 1/2 EGCG	2083.6 ± 783.88 #
HC + 1/2 EC	2103.8 ± 647.97 #
HC + 1/2 ECG	2001.1 ± 592.17 #

HC: high-calorie-fed mice; EGC: epigallocatechin; EGCG: epigallocatechin gallate; EC: epicatechin; ECG: epicatechin gallate. #: *p* < 0.05 vs. age-matched control value. *: *p* < 0.05 vs. age-matched HC value.

**Table 5:** Blood biochemistry values of mice in each group.

	**Glucose (mg/dL)**	**Total Protein (g/dL)**	**Total Lipid (mg/dL)**	**LDL-Cholesterol (mg/dL)**
HC + Green Tea	208.4 ± 21.40 #	5.28 ± 0.31 #	410.3 ± 39.88 #	8.74 ± 1.63 #
HC + 1/2Green Tea	210.4 ± 21.85 #	5.44 ± 0.42 #	436.3 ± 45.82 #	8.87 ± 1.52 #
HC + β-cryptoxanthin	223.4 ± 19.46 #	5.79 ± 0.38 #	449.2 ± 46.38 #	9.01 ± 1.87 #
HC + 1/2 EGC	231.5 ± 24.87 #	5.92 ± 0.34 #	439.0 ± 38.29 #	8.46 ± 1.93 #
HC + 1/2 EGCG	232.5 ± 22.71 #	5.71 ± 0.42 #	421.7 ± 37.87 #	8.43 ± 1.38 #
HC + 1/2 EC	229.8 ± 20.01 #	5.94 ± 0.42 #	428.9 ± 43.44 #	8.72 ± 1.68 #
HC + 1/2 ECG	238.9 ± 19.78 #	5.96 ± 0.35 #	436.6 ± 39.80 #	8.59 ± 1.55 #
	**HDL-Cholesterol (mg/dL)**	**Free-Cholesterol (mg/dL)**	**NEFA (mEq/L)**	**Triglyceride (mg/dL)**
HC + Green Tea	59.9 ± 8.64 #	39.7 ± 3.88 #	1658.4 ± 120.37 #	90.01 ± 13.77 #
HC + 1/2Green Tea	59.9 ± 6.92 #	40.4 ± 3.02 #	1763.4 ± 105.55 #	91.98 ± 14.89 #
HC + β-cryptoxanthin	61.4 ± 5.74 #	42.0 ± 3.21 #	1759.5 ± 123.97 #	92.02 ± 15.88 #
HC + 1/2 EGC	58.8 ± 6.37 #	42.0 ± 3.09 #	1698.9 ± 128.34 #	90.85 ± 13.89 #
HC + 1/2 EGCG	59.4 ± 8.83 #	39.5 ± 3.24 #	1735.8 ± 104.76 #	91.74 ± 13.85 #
HC + 1/2 EC	61.3 ± 9.35 #	42.3 ± 3.89 #	1719.7 ± 119.33 #	90.93 ± 15.03 #
HC + 1/2 ECG	62.1 ± 8.00 #	42.9 ± 3.16 #	1692.5 ± 113.79 #	91.65 ± 14.58 #

HC: high-calorie-fed mice; EGC: epigallocatechin; EGCG: epigallocatechin gallate; EC: epicatechin; ECG: epicatechin gallate. #: *p* < 0.05 vs. age-matched control value.

**Table 6:** Blood biochemistry values of mice in each group.

	Alkaline Phosphatase (IU/L)	AST (IU/L)	ALT (IU/L)
HC + Green Tea	553.8 ± 59.43 #	1438.3 ± 139.21 #	793.32 ± 138.25 #
HC + 1/2Green Tea	569.2 ± 46.39 #	1592.9 ± 148.89 #	804.85 ± 148.12 #
HC + β-cryptoxanthin	583.9 ± 49.21 #	1629.4 ± 148.81 #	822.96 ± 131.09 #
HC + 1/2 EGC	592.5 ± 38.39 #	1673.7 ± 134.27 #	808.29 ± 118.92 #
HC + 1/2 EGCG	583.2 ± 38.44 #	1539.3 ± 129.99 #	797.74 ± 142.26 #
HC + 1/2 EC	591.2 ± 40.39 #	1577.5 ± 151.49 #	806.17 ± 123.47 #
HC + 1/2 ECG	584.7 ± 55.24 #	1502.1 ± 137.74 #	818.08 ± 139.01 #

HC: high-calorie-fed mice; EGC: epigallocatechin; EGCG: epigallocatechin gallate; EC: epicatechin; ECG: epicatechin gallate. #: *p* < 0.05 vs. age-matched control value.

## Data Availability

The datasets used and/or analyzed during this study are available from the corresponding author upon reasonable request.
